# Isolated Bilateral Cerebellar Dysfunction as the Initial Manifestation of HIV Infection: A Diagnostic Challenge, Case Report, and Literature Review

**DOI:** 10.1007/s12311-025-01861-8

**Published:** 2025-06-18

**Authors:** Ritwick Mondal, Shramana Deb, Ananya Sengupta, Subhadeep Banerjee, Nirmalya Ray, Mona Tiwari, Jayanta Roy, Julián Benito-León

**Affiliations:** 1 Department of Neurology, Manipal Groups of Hospitals, Mukundapur, Kolkata India; 2 Department of Neuroradiology, Institute of Neurosciences, Kolkata, India; 3 Department of Neurology, 12 de Octubre University Hospital, Madrid, Spain; 4 Group of Neurodegenerative Diseases, Hospital Universitario 12 de Octubre Research Institute (imas12), Madrid, Spain; 5 Faculty of Medicine, Complutense University of Madrid, Madrid, Spain

**Keywords:** HIV-1, HIV-2, Cerebellar ataxia, Isolated cerebellar dysfunction, Rhombencephalitis, JC virus, Granule cell neuronopathy, HIV-associated neurological complications, Antiretroviral therapy

## Abstract

Despite widespread access to antiretroviral therapy, neurological complications remain common in people living with human immunodeficiency virus (HIV). While opportunistic infections and HIV-associated malignancies are the usual causes, there is growing recognition of atypical, non-opportunistic neurological syndromes as early manifestations of HIV infection—even in the absence of prior diagnosis or treatment. We report the case of a 46-year-old woman with type 2 diabetes mellitus and a history of pulmonary tuberculosis who presented with a two-month history of progressive unsteady gait, dysarthria, and frequent falls. Neurological examination revealed isolated cerebellar dysfunction. Brain magnetic resonance imaging showed T2 and FLAIR hyperintensities in the bilateral middle cerebellar peduncles and adjacent white matter, suggestive of rhombencephalitis. Extensive diagnostic work-up for infectious, autoimmune, and paraneoplastic etiologies was negative. HIV testing revealed dual seropositivity for HIV-1 and HIV-2, with undetectable HIV-1 RNA and a low CD4 + T-cell count. The patient improved clinically with supportive care and was referred for antiretroviral therapy. At the two-month follow-up, she demonstrated marked recovery. Although cerebellar involvement in HIV is typically associated with opportunistic infections or neoplasia, this case illustrates that bilateral cerebellar dysfunction can represent the first clinical manifestation of HIV infection. We also review previously reported cases in which cerebellar signs were the initial presentation, emphasizing the need to consider HIV testing in patients with unexplained cerebellar syndromes.

## Background

Human immunodeficiency virus (HIV) remains a major global health concern, with over 3 million people living with HIV/AIDS in India as of 2024 and an adult prevalence of 0.21%.[1] Despite advances in combined antiretroviral therapy, neurological symptoms are the initial presentation in approximately 10% of newly diagnosed cases,[2] with nearly two-thirds linked to opportunistic infections or HIV-associated malignancies, particularly in individuals with advanced immunosuppression (CD4 < 200 cells/mm³) [3].

The clinical course of HIV has transformed in the combined antiretroviral therapy era,[4] with a marked decline in the incidence of opportunistic infections and a shift toward chronic, non-opportunistic complications. Paradoxically, the prevalence of HIV-associated neurocognitive disorders has remained stable or increased, likely reflecting longer survival and aging of the HIV-infected population.[5] Moreover, the proportion of patients presenting with central nervous system involvement at diagnosis has plateaued since 2003, underscoring persistent gaps in early detection.[6] Finally, dual infection with HIV-1 and HIV-2—although uncommon—carries distinct diagnostic and therapeutic implications and may contribute to atypical neurological manifestations.[7].

Ataxia is the second most common movement disorder in people living with HIV/AIDS, following parkinsonism. It is most often attributed to opportunistic infections, particularly progressive multifocal leukoencephalopathy, a demyelinating disease caused by JC virus reactivation in the central nervous system.[8] In the post-combined antiretroviral therapy era, efavirenz has also emerged as a potential cause of late-onset cerebellar ataxia and encephalopathy, particularly in patients with supratherapeutic plasma levels.[9] Additionally, JC virus granule cell neuronopathy (JCV-GCN)—a distinct entity marked by progressive cerebellar atrophy due to lytic infection of cerebellar granule neurons—has been increasingly recognized in both HIV-seropositive individuals and other immunosuppressed populations, even in the absence of classical progressive multifocal leukoencephalopathy lesions.[10].

We report the clinical course of a newly diagnosed HIV-positive adult woman who presented with isolated, acute-onset, bilaterally progressive cerebellar ataxia as the first manifestation of HIV infection—without evidence of opportunistic infection or prior antiretroviral therapy—and review similar cases in which cerebellar dysfunction was the initial clinical presentation of HIV.

### Case Presentation

A 46-year-old woman with a history of type 2 diabetes mellitus and no known arterial hypertension presented with a two-month history of sudden-onset, rapidly progressive unsteady gait, imbalance, dysarthria, and bilateral limb incoordination, resulting in multiple falls. She denied seizures, loss of consciousness, head trauma, anorexia, facial asymmetry, headache, or bowel or bladder incontinence.

Her past medical history was notable for pulmonary tuberculosis, treated successfully 20 years earlier. Apart from occasional alcohol consumption, her medical history was otherwise unremarkable.

She had been in her usual state of health until mid-November 2024, when she began to experience progressive gait instability, frequent falls, and increasing difficulty with speech articulation.

On admission, her general physical examination was normal, and her cognitive function was intact. Neurological examination revealed no sensory or motor deficits. Muscle strength was preserved (MRC grade 5/5 in all limbs), but mild hypotonia was present in the lower extremities. Plantar responses were flexor bilaterally. Cranial nerve examination revealed dysarthric speech, characterized by poor articulation and blurred consonant pronunciation. Cerebellar signs were prominent, including broad-based gait requiring two-person assistance, dysdiadochokinesia, past-pointing, intention tremor, and a positive heel-knee-shin test—all bilaterally. The fundoscopy was normal. Her Scale for the Assessment and Rating of Ataxia (SARA) score was 27, and the Modified Rankin Scale (mRS) score was 4.

**Fig. 1:**
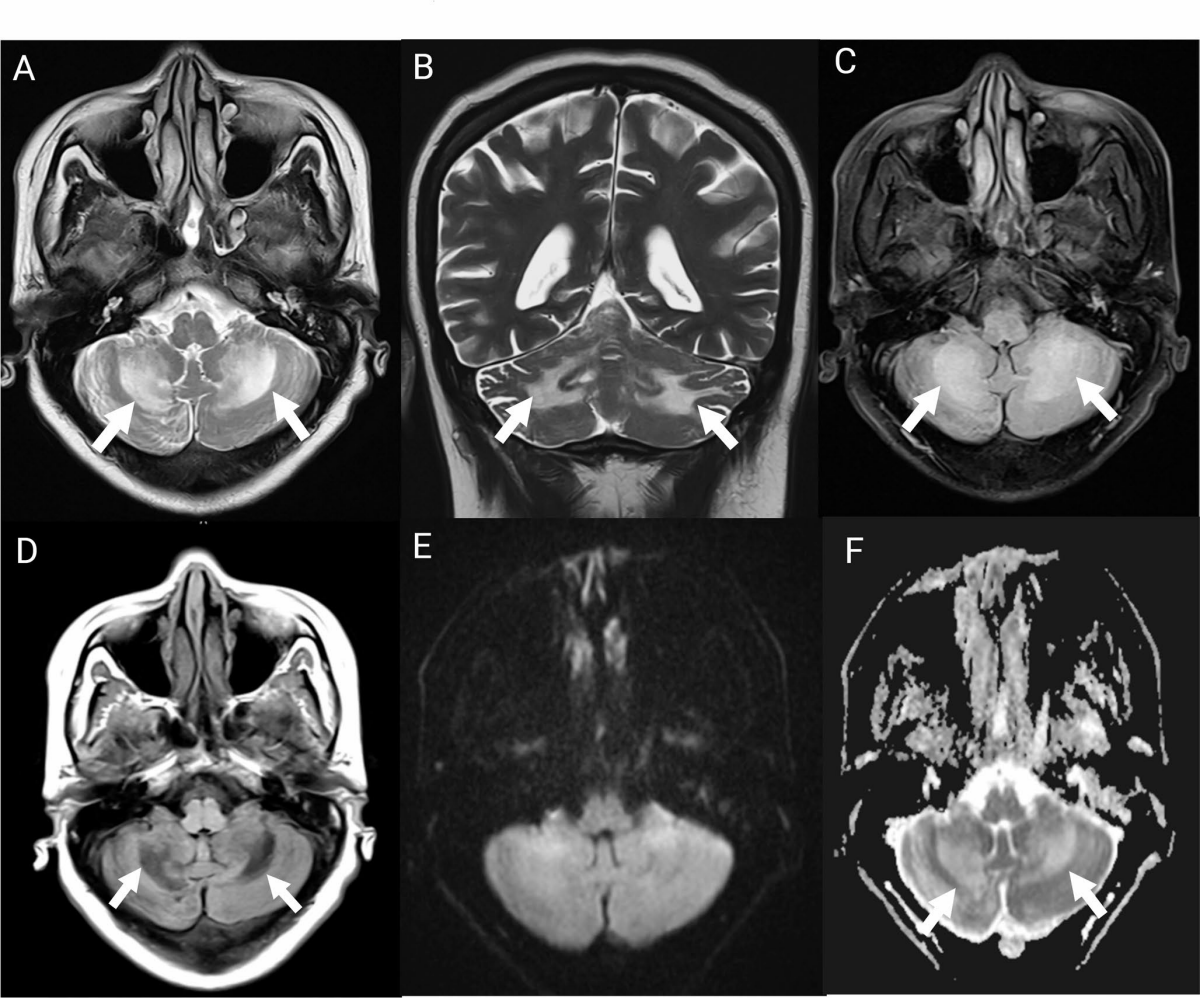
Magnetic resonance imaging of the brain. (**A**, **B**) Axial and coronal T2-weighted images show symmetrical hyperintensities involving the bilateral middle cerebellar peduncles and adjacent cerebellar white matter (white arrows). (**C**) Axial FLAIR image reveals similar hyperintensities. (**D**) Axial T1-weighted image demonstrates corresponding hypointensities. (**E**) Diffusion-weighted imaging (b = 1000 s/mm²) does not show restricted diffusion. (**F**) The apparent diffusion coefficient (ADC) map shows hyperintensity in the same regions, suggesting the T2 shine-through effect and excluding true diffusion restriction

She was admitted to the neurology unit for further evaluation. Initial serum and cerebrospinal fluid (CSF) biochemical and microscopic analyses were unremarkable (Table 1). Neuroimaging studies, including non-contrast computed tomography and magnetic resonance imaging (MRI) of the spine, revealed no acute abnormalities. Brain MRI demonstrated symmetrical areas of T2/FLAIR hyperintensity and corresponding T1 hypointensity, without diffusion restriction—findings suggestive of rhombencephalitis of either infectious or autoimmune etiology (Fig. 1). Importantly, there was no evidence of cerebral or cerebellar atrophy. Magnetic resonance angiography was normal. Additional tests, including visual-evoked potentials and brainstem auditory evoked responses, yielded normal results. Chest radiography and abdominal ultrasonography were also unremarkable.

**Table 1:** Baseline clinical, laboratory, and differential diagnostic findings

**A) Clinical Features**	
**Parameter**	**Finding**
Gait	Broad-based, unsteady
Speech	Dysarthria
Coordination	Bilateral dysmetria
Sensory examination	Normal
Cognition	Intact
Motor strength	Normal in all limbs
Reflexes	Normal; plantar responses flexor
Scale for the Assessment and Rating of Ataxia (SARA)	27 at baseline, improved to 18
Modified Rankin Scale (mRS) score	4 at baseline, improved to 3
**B) Instrumental and Laboratory Findings**	
**Test/Parameter**	**Result**
CD4 count	CD4 + count (147 cells/µL; 23.05%) with a normal CD4/CD8 ratio (0.50) and an absolute CD8 + count of 285 cells/µL (46.23%)
HIV-1 RNA viral load	Undetectable
HIV-2 RNA viral load	Less than 100 IU/mL
Autoimmune panel	Negative serum panel (anti-NMDA-R, AMPAR1/2, CASPR2, LGI1, GABA-B1/B2 antibodies)
Testing for infections	Negative serologies: antibodies against cytomegalovirus (both IgG and IgM), *Treponema pallidum* hemagglutination assay, fluorescent treponemal antibody absorption test, antibodies against *Orientia tsutsugamushi*, venereal disease research laboratory test, varicella IgM, and toxoplasma IgM
	Positive serology: toxoplasma IgG at 2.23 IU, suggestive of prior exposure
	Cerebrospinal fluid multiplex polymerase chain reaction panel (Biofire): negative for 15 pathogens, including cryptococcal antigen, cytomegalovirus, Epstein–Barr virus, *Orientia tsutsugamushi*, and JC virus
	Cerebrospinal fluid testing with GeneXpert (for tuberculosis), venereal disease research laboratory test, adenosine deaminase, acid-fast bacilli staining, Gram staining, and India ink preparation: all results were within normal limits or unremarkable.
Cerebrospinal Fluid	Normal protein and glucose; no pleocytosis; negative JC virus polymerase chain reaction
Brain magnetic resonance imaging	Bilateral T2/FLAIR hyperintensities in middle cerebellar peduncles; no contrast enhancement
Electroencephalography	Normal
Visual-evoked potentials	Normal
Brainstem-evoked response audiometry	Normal
**C) Differential Diagnosis**	
*Toxic etiopathology*	Ruled out based on history and unremarkable bloodwork (no evidence of alcohol intoxication).
*Drug-induced cerebellopathy*	There was no history of prior antiretroviral therapy or other medications.
*Metabolic or nutritional cerebellopathy* (e.g., thiamine or vitamin E deficiency)	Ruled out by normal laboratory and dietary history.
Cerebellar infarcts or structural lesions	Excluded by MRI findings (no diffusion restriction or mass effect).
*Opportunistic infections (serum)*	There was no evidence of active infection; positive toxoplasma IgG was consistent with past exposure.
*Opportunistic infections (cerebrospinal fluid)*	There was no evidence of central nervous system infection.
*Paraneoplastic syndromes*	Negative anti-neuronal antibody panel.
*Antiretroviral therapy-related*	There was no prior exposure.
*Hereditary or familial ataxia*	There was no family history of spinocerebellar ataxia or progressive ataxia among first- or second-degree relatives.
*Progressive multifocal leukoencephalopathy*	Cerebrospinal fluid negative for JC virus; a clinical improvement over time argues against diagnosis.
*Autoimmune encephalitis*	Negative serum panel.
*Central nervous system tuberculosis*	Cerebrospinal fluid GeneXpert negative; adenosine deaminase level within normal range.
*JC virus granule cell neuronopathy*	It cannot be confirmed in the absence of cerebellar biopsy; however, negative JC virus polymerase chain reaction in cerebrospinal fluid, clinical improvement, and absence of cerebellar atrophy make it less likely.

Given the imaging findings, a second lumbar puncture was performed to investigate possible autoimmune, paraneoplastic, or infectious etiologies. Pending results, intravenous immunoglobulin was initiated at 2 g/kg based on a presumed diagnosis of immune-mediated cerebellitis. However, no clinical improvement was observed.

Further testing by ELISA confirmed HIV seropositivity for both type 1 and type 2. Quantitative polymerase chain reaction (PCR) revealed undetectable HIV-1 RNA and a low HIV-2 RNA viral load (< 100 IU/mL). Flow cytometry (BD-FACS Canto II) showed a reduced absolute CD4 + count (147 cells/µL; 23.05%) with a normal CD4/CD8 ratio (0.50) and an absolute CD8 + count of 285 cells/µL (46.23%). CSF multiplex PCR (BioFire) was negative for 15 pathogens, including cryptococcal antigen, JC virus, Epstein–Barr virus, and cytomegalovirus (CMV). CSF Venereal Disease Research Laboratory (VDRL), adenosine deaminase, GeneXpert, Gram stain, acid-fast bacilli stain, and India ink stain were all negative. Serum testing also yielded negative results for anti-CMV IgG/IgM, treponema pallidum hemagglutination assay (TPHA), fluorescent treponemal antibody absorption (FTA-ABS) test, scrub typhus IgM/IgG, Venereal Disease Research Laboratory (VDRL), varicella IgM, and anti-toxoplasma IgM. However, anti-Toxoplasma IgG was positive (2.23 IU), suggesting past exposure (Table 1). She was empirically treated with sulfadiazine (500 mg daily), pyrimethamine (50 mg daily), and folinic acid (15 mg daily). Vitamin B12, vitamin E, and serum protein electrophoresis were all within normal limits.

In parallel with pharmacologic management, she was initiated on neurorehabilitation and speech therapy. After 8 days of hospitalization, she showed marked improvement in speech and upper limb coordination, although her gait remained broad-based and ataxic. She was discharged in stable condition with improved scores (mRS: 3; Scale for the Assessment and Rating of Ataxia [SARA]: 22) and referred to the local antiretroviral therapy center for initiation of combination antiretroviral therapy.

At the two-month follow-up, the patient showed significant clinical improvement, with progressive resolution of dysarthria and ataxia. Her SARA score improved to 18, with a stable mRS of 3, indicating a favorable response to symptomatic treatment and combined antiretroviral therapy.

## Discussion

This report describes a rare case of acute-onset, rapidly progressive cerebellar syndrome as the initial clinical manifestation of HIV-1 and HIV-2 co-infection. It raises an important question: why do some individuals present with isolated cerebellar dysfunction in the absence of opportunistic infections or prior antiretroviral therapy? Our case contributes to the limited number of reports describing HIV-associated cerebellar dysfunction as a primary presentation of HIV infection, occurring without any identifiable infectious, neoplastic, paraneoplastic, toxic, drug-induced, or autoimmune etiology (Table 1).[11–17].

Although HIV is known to cause ataxia, it is typically attributed to secondary mechanisms, particularly opportunistic infections. A cerebellar syndrome that is acute, rapidly progressive, and directly attributable to HIV itself—as observed in our case—is exceptionally rare. Through an extensive review of the literature, we identified 10 previously reported cases in which isolated cerebellar involvement represented the initial manifestation of HIV infection, occurring in the absence of any identifiable secondary cause (Table 2). Among these, only two mirrored the acute and rapidly evolving clinical presentation seen in our patient.[13, 17].

**Table 2:** Summary of reported cases of isolated cerebellar dysfunction as the initial manifestation of HIV infection in the absence of opportunistic infections

Case / First Author (Year)	Age / Sex	Neurological Presentation at Onset	Progression Before Admission	HIV Status at Diagnosis	HIV Type	CD4 Count	CSF Findings	MRI Findings	Outcome
Tagliati et al. (1998) [11]	27–57 / 8 Male, 2 Female	Gait ataxia (universal), variably associated with dysarthria, dysmetria, nystagmus, or truncal instability	Subacute in 7, chronic in 3	Known HIV-positive in 7; cerebellar syndrome as the first manifestation of HIV in 3	HIV-1	≤ 200 cells/mm³ in 5 of 9 cases with available data	Unrevealing in most cases, one patient had elevated immunoglobulin G index and the presence of oligoclonal bands.	Cerebellar atrophy (mild to severe) in 7 of 9 cases with available data; cerebral atrophy in 6 of 9; white matter abnormalities in 3 of 9; no mass lesions reported.	Progressive; no improvement
Puertas et al. (2003) [12]	31 / Female	Gait ataxia, difficulty with fine movements, mild weight loss, dysarthria, bilateral upper limb dysmetria, dysdiadochokinesia, horizontal nystagmus	2-year history of ataxia	Unknown	HIV-1	18 cells/mm³	Elevated CSF IgG, oligoclonal bands	Severe cerebellar atrophy	No improvement at 2 months
Mishra et al. (2017) [13]	18 / Male	Broad-based gait, tremor, hypotonia in all limbs, dysarthria, nystagmus, dysmetria	3-month history of gait disturbance and tremor	Unknown	HIV-1	26 cells/mm³	10 lymphocytes/µL; protein: 41 mg/dL; glucose: 55 mg/dL	Severe cerebellar atrophy	Improved with rehabilitation
Hoyer et al. (2017) [14]	35 / Male	Broad-based gait, dysmetria, dysdiadochokinesia, dysarthria, slow saccades, tremor (SARA: 11.5)	Symptoms for over 2 years	Unknown	HIV-1	33.3% CD4; CD4/CD8 ratio: 0.5	Pleocytosis (98 cells/µL), positive oligoclonal bands	Cerebellar atrophy; no lesions; mild cerebral atrophy	No change after 3 months (SARA: 11.5)
Pedroso et al. (2018) [15]	44 / Female	Cerebellar ataxia, dysmetria, dysdiadochokinesia, dysarthria, saccadic eye movements, nystagmus	1-year history of gait ataxia	Unknown	HIV-1	65 cells/mm³	Protein: 51.9 mg/dL; glucose: 40 mg/dL	Mild cerebellar atrophy	Mild improvement at 6 months
	47 / Male	Bilateral dysmetria, dysdiadochokinesia, ataxic gait	10-year history of dysarthria and gait ataxia	Unknown	HIV-1	827 cells/mm³	Unremarkable	Global cerebellar atrophy	No improvement at 3 years
Briskyan et al. (2023) [16]	52 / Male	Cerebellar ataxia, oculomotor dysfunction, scanning speech	1-year history of gait ataxia	Unknown	HIV-1	Not reported	Unremarkable	Mild cerebellar atrophy	No improvement
Jain et al. (2023) [17]	40 / Male	Gait ataxia, dysarthria, tremor, dysmetric saccades	3-month history of ataxia	Unknown	HIV-1	450 cells/mm³	Unremarkable	Mild pancerebellar atrophy	Subtle improvement at 1 month

While HIV is known to exert widespread effects on the central nervous system, the specific regional and cellular targets remain incompletely understood. It is still unclear whether the neuropathological changes observed in HIV-associated neurological disorders stem from direct viral persistence within the brain, the neurotoxic effects of viral proteins or nucleic acids, or sustained immune-mediated neuroinflammation. Notably, HIV-associated neurocognitive disorders and dementia remain prevalent—even among individuals with suppressed viral loads on effective combined antiretroviral therapy.[18, 19] These findings support the hypothesis that the brain may act as a reservoir for persistent or cryptic HIV infection, permitting low-level replication despite systemic viral control. Neuropathological studies have increasingly implicated microglia and macrophages as key targets, with these long-lived cells potentially harboring latent virus and sustaining chronic neuroinflammation.[20] A recent study by Yang et al. [21] further reinforced this model, demonstrating widespread transcriptomic and epigenetic alterations across multiple brain cell types in individuals living with HIV.

The pathophysiology of isolated bilateral cerebellar ataxia directly attributable to HIV infection—without secondary causes such as opportunistic infections or toxins—remains poorly understood.[15] Proposed explanations include immune-mediated injury and direct HIV-induced neurodegeneration. Histopathological studies have demonstrated cerebellar white matter and cortical degeneration in the absence of opportunistic infections, pointing toward a direct neurotoxic effect of the virus.[22] Notably, several HIV envelope proteins—such as gp120, gp41, and Tat—have been implicated in neuronal damage, particularly within the cerebellar dentate and inferior olivary nuclei.[23] In addition, the viral accessory protein Vpr has been shown to induce apoptosis in cerebellar neurons, further supporting a pathogenic role for direct viral toxicity.[24].

Notably, our patient’s MRI showed bilateral T2 and FLAIR hyperintensities with corresponding T1-weighted hypointensities in the middle cerebellar peduncles—findings that initially raised concern for progressive multifocal leukoencephalopathy. Progressive multifocal leukoencephalopathy typically occurs in individuals with severe cellular immunosuppression.[10, 25] Although CSF PCR for JC virus was negative, this does not definitively exclude the diagnosis, as false-negative results may occur.[10, 25, 26] Brain biopsy, the diagnostic gold standard,[26] was not pursued. The patient’s marked clinical improvement after starting combination antiretroviral therapy—currently the cornerstone of management in such cases—further supports progressive multifocal leukoencephalopathy as a plausible underlying cause despite the absence of definitive confirmation.

An additional consideration is JCV-GCN, a distinct entity characterized by selective infection of cerebellar granule neurons, which may manifest with progressive cerebellar ataxia in the absence of typical progressive multifocal leukoencephalopathy lesions.[10] As highlighted in a recent review, JCV-GCN is increasingly recognized among immunocompromised individuals, including those with HIV.[10] Notably, none of the previously reported cases summarized in Table 2 include sufficient virological or histopathological evaluation to confirm or exclude this condition—with the partial exception of Tagliati et al.,[11] who identified JCV DNA in cerebellar tissue and granule cell loss in one patient, and reported granule cell loss in a second, both findings consistent with what is now recognized as JCV-GCN. However, the entity had not yet been formally defined at the time. In our case, while CSF PCR for JCV was negative and the clinical improvement without evidence of cerebellar atrophy argues against JCV-GCN, a definitive exclusion would require neuropathological confirmation.

One of the key distinguishing features of the present case is the absence of cerebellar atrophy, which was present in nearly all previously reported cases of isolated cerebellar dysfunction associated with HIV (Table 2). MRI in our patient showed signal changes in the bilateral middle cerebellar peduncles without evidence of atrophy in the cerebellum or cerebral cortex, a finding that distinguishes our case from nearly all previously reported cases. This is particularly relevant given that cerebellar atrophy—often progressive—is a hallmark of HIV-associated cerebellar syndromes (Table 2). Furthermore, unlike most previous cases, which showed minimal or no response to treatment, our patient demonstrated marked clinical improvement over time. This favorable trajectory, in conjunction with the extensive exclusion of secondary causes, strongly supports a primary, non-opportunistic HIV-related mechanism affecting cerebellar pathways. Thus, this case underscores the heterogeneity of cerebellar involvement in HIV and highlights the importance of considering early-stage, potentially reversible cerebellar dysfunction in newly diagnosed patients—especially in the absence of atrophy or opportunistic disease.

An unexpected finding in our case was the dissociation between the patient’s undetectable HIV-1 RNA and persistently low CD4 + T-lymphocyte count despite the absence of antiretroviral therapy. Although uncommon, this immunovirological profile has been described in certain individuals with long-standing HIV infection, particularly elite or viremic controllers, where immunologic decline may occur independently of plasma viremia. Mechanisms proposed to explain this immunovirological dissociation include immune exhaustion, ongoing low-level viral replication potentially occurring in tissue compartments, and sustained immune activation with elevated proinflammatory cytokines despite undetectable plasma HIV-1 RNA.[27] In our patient, dual seropositivity for HIV-1 and HIV-2 may further contribute to this atypical immune trajectory, as HIV-2 is known for low or undetectable plasma viral load and a more indolent course, sometimes resulting in late-onset immunodeficiency.[28].

The management of HIV-related movement disorders remains challenging, with limited high-quality evidence to guide treatment. While chorea may respond to targeted interventions—including low-dose dopamine receptor antagonists in selected cases, or treatment of underlying infections—other manifestations, such as parkinsonism, tremor, and ataxia, often respond poorly to standard therapies [8]. Encouragingly, our patient demonstrated gradual clinical improvement with symptomatic management and combined antiretroviral therapy.

In conclusion, cerebellar involvement in HIV is mostly attributed to opportunistic infections or central nervous system lymphoma. However, isolated cerebellar dysfunction as the initial manifestation of HIV infection, particularly in the absence of secondary causes, remains rare and underrecognized in clinical practice. This case underscores the importance of considering HIV in the differential diagnosis of adults presenting with unexplained, progressive cerebellar ataxia.

A deeper understanding of the underlying mechanisms—whether related to neuroimmune dysregulation, direct neurotoxic effects of viral proteins, or HIV-mediated cerebellar neurodegeneration—is essential. Our findings, supported by a review of the limited literature, highlight the need for heightened clinical suspicion and further investigation into the neuropathological consequences of both HIV-1 and HIV-2 infections in the cerebellum.

## Data Availability

No datasets were generated or analysed during the current study.
